# Defining workload components and activity standards for human resources professionals in Iranian public hospitals: A WISN-based framework for strengthening non-clinical workforce planning

**DOI:** 10.1371/journal.pone.0356051

**Published:** 2026-10-08

**Authors:** Robabeh Emrouzi, Ali Janati, Masoumeh Gholizadeh, Mahmood Yousefi, Sajjad Ahmadi

**Affiliations:** 1 School of Management and Medical Informatics, Tabriz University of Medical Sciences, Tabriz, Iran; 2 Iranian Center of Excellence in Health Management, School of Management and Medical Informatics, Tabriz University of Medical Sciences, Tabriz, Iran; 3 Department of Health Economics, School of Management and Medical Informatics, Tabriz University of Medical Sciences, Tabriz, Iran; 4 Emergency and Trauma Care Research Center, Tabriz University of Medical Sciences, Tabriz, Iran; Khoy University of Medical Sciences, IRAN, ISLAMIC REPUBLIC OF

## Abstract

**Background:**

Defining valid workload components and activity standards is a prerequisite for workload-based workforce planning. This study aimed to develop and validate workload components and activity standards for human resources (HR) departments in Iranian public hospitals using a framework aligned with the Workload Indicators of Staffing Need (WISN) methodology.

**Methods:**

A consensus-based mixed-method approach was used. An initial list of HR activities was developed through expert consultation and enriched through approximately 200 hours of field observation and staff interviews in a selected public hospital. The resulting workload components and preliminary activity standards were assessed using a structured Delphi questionnaire based on WISN principles. Twenty-three HR and workforce-planning experts participated in two Delphi rounds. Consensus was defined as ≥70% agreement on a five-point Likert scale.

**Results:**

At the end of the second round of the Delphi study, 91.6% agreement was achieved. A total of 76 workload components for HR staff were identified and categorized into service, support, and additional activities. The most notable changes from the initial list and the highest level of expert disagreement were related to strategic tasks for the job group, unobservable and cognitive work, repetitive activities, and the allocation of activities in WISN activity categories.

**Conclusion:**

The study developed a validated and context-specific set of workload components and activity standards for hospital HR departments. The findings provide a practical foundation for applying the WISN methodology to non-clinical staff and support more accurate workforce planning, organizational redesign, and evidence-based management of human resources in hospitals.

## Introduction

The World Health Organization (WHO) promotes workforce planning grounded in evidence as a leading approach to optimizing global health system performance and providing equitable health service access internationally [1,2]. Continuous improvements to health workforce management have not resolved imbalances in worker distribution, shortages, and increasing demands for services that many countries face. Eastern Mediterranean Region countries have addressed these challenges with an array of approaches to advance health workforce development and planning [3]. Similar challenges are evident across health systems in the region, where growing demand for healthcare services and resource constraints place increasing pressure on workforce management and service delivery [3–5]. These challenges affect both clinical and non-clinical personnel, whose coordinated contributions are essential for maintaining effective healthcare organizations [6].

In Iran, workforce-related challenges remain a significant concern. Although comprehensive health workforce strategies have been introduced, hospitals continue to experience imbalances in the distribution of clinical and non-clinical staff which may reduce organizational efficiency and hinder service optimization [7,8]. Given the complexity of human resource challenges in the Iranian health system, strengthening evidence-based workforce planning is a fundamental priority [9].

While considerable attention has been devoted to estimating workforce requirements for traditional clinical roles, such as physicians and nurses, non-clinical positions have been largely neglected [10]. This neglect reflects a broader gap in global health workforce planning, as non-clinical staff, exemplified by over half a million National Health Service employees in England, are essential to healthcare performance [11]. Effective healthcare delivery depends on both clinical expertise and a well-integrated network of supportive services that ensure smooth operations and positive outcomes [4,12,13]. Appropriate recruitment of non-clinical staff enables clinical staff to focus more on specialized care, thereby increasing overall team performance [14,15]. Some broad studies have included non-clinical positions as secondary components. The characteristics of multitasking, irregular duty cycles, and the lack of standardized activities in non-clinical positions complicate accurate workforce estimation in this field [14,16].

Among non-clinical positions, the human resources department, as a key strategic department, plays a pivotal role in aligning efforts with the broader goals of the organization, and the proper functioning and standards of human resources professionals are critical [17]. The performance of HR management has been associated with increased patient satisfaction, reduced medical errors, and improved overall productivity [18]. Despite numerous changes and reforms over the past few decades to improve hospital processes, the role of the human resources group is still often ignored [19]. These roles that enable clinical functions are often managed without evidence-based staffing norms, creating a critical vulnerability in the health system [11,20].

The WISN approach, endorsed by the WHO, is a prominent workload-based tool for calculating staffing needs [21]. The method consists of eight steps, with steps 3 (identifying workload components) and 4 (defining activity standards) being foundational, as they determine which tasks are performed and how much time is needed to complete them under local conditions [21–23]. A variety of methods, including literature reviews, interviews, direct observation, and focus group discussions, have been used to collect data for stages 3 and 4 in various WISN studies [24–31]. The accuracy of these initial steps enables more precise workforce estimations [22,32,33]. Lists of workload components (WC) and activity standards (AS) are not readily available in most countries [34]. Most WISN studies emphasize the final number of employees, while the methodological rigour of these initial steps is not sufficiently clear and well documented, especially for non-clinical positions [32,34].

Although WISN applies to all personnel types, non-clinical roles remain underrepresented in most WISN applications [16,35]. The lack of a valid and agreed-upon set of workload components and activity standards for human resource (HR) departments, as well as a clear framework for generating such data, is evident in Iranian public hospitals. This gap limits the capacity for evidence-based workforce planning in administrative areas. Therefore, the present study aimed to develop and validate workload components and activity standards for hospital HR departments using a WISN-based framework. A structured and context-sensitive approach is presented to support workforce planning in non-clinical hospital positions. The findings contribute to broader national efforts to strengthen evidence-based estimates of staffing.

## Materials and methods

### Overall study design and WISN adaptation

This study aimed to develop baseline workload components(WC) and activity standards(AS) for HR departments in Iranian public hospitals by following steps three and four of the WISN methodology. This study consists of four phases: 1. developing a preliminary activity list by interviewing experts, 2. process mapping through field observation, 3. consensus development using the Delphi technique, and 4. synthesis of validated workload components and activity standards. Within the Workload Indicator of Staffing Needs (WISN) framework, workload components (WC) are the tasks undertaken on a routine basis by a particular staff category, and activity standards (AS) are the time that a diligent and well-trained staff member takes to carry out each task to the satisfaction of the professional standards. This study examines step 3 and 4 of the WISN methodology: component identification and activity standardization. Since the HR processes involve the administration of varying process-driven and cognitive tasks that are not aligned with the normal clinical services, certain adjustments of the methodology were utilized to achieve greater activity and workload measurement within the framework of the WHO WISN. Core methodological elements, including the classification of workload components, activity standard development, Delphi-based validation, and consensus criteria, were guided by the WHO WISN methodology.

The study was carried out between October 2023 to October 2024. The development process involved a comprehensive literature review, an analysis of strengths and weaknesses, recommendations from previous WISN implementation experiences, and a review of national documents, job descriptions, interviews with national stakeholders, and field observations, which together generated richer insights [21,36,37].The overall methodology framework is adapted from a structured consensus development model [37], and key adaptations consistent with the nature of the administration’s work were applied. Fig 1 provides a schematic overview of the integrated process.

**Fig 1:**
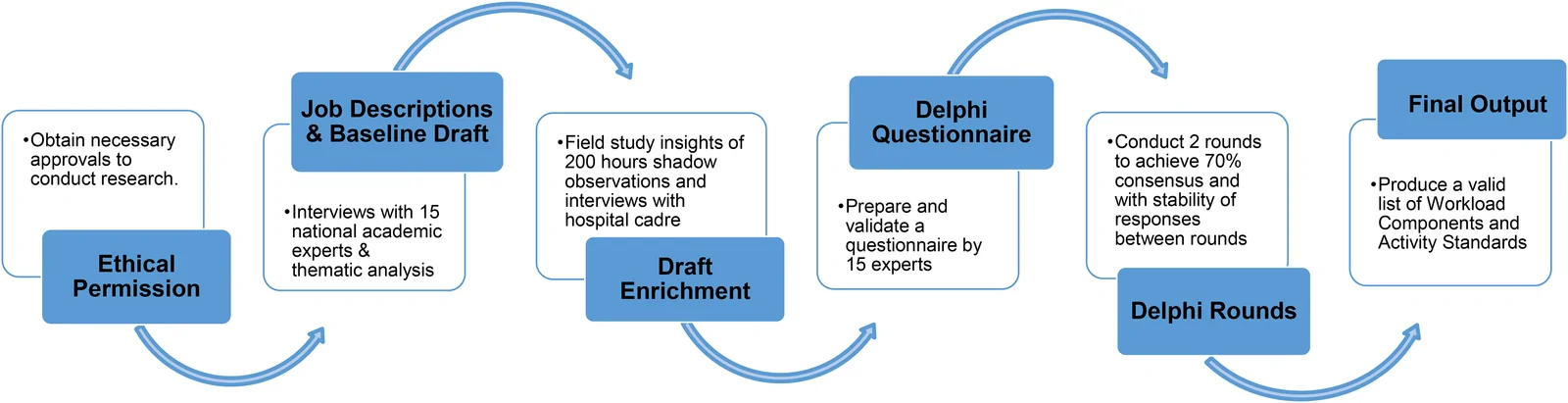
Schematic overview of the methodology used to develop workload components and activity standards for HR departments.

The present study focused on WISN steps 3 and 4, namely the identification of workload components and establishment of activity standards. These outputs provide essential inputs for subsequent WISN calculations, including the estimation of Category Allowance Standards (CAS) and Individual Allowance Standards (IAS). However, calculation of CAS and IAS requires additional information, particularly updated Available Working Time (AWT) data, which was not available within the scope of the present study. Therefore, the study was limited to developing and validating workload components and activity standards for future workforce estimation exercises.

### Data collection procedures

The first step was to answer the question: “What do HR staff actually do?” Semi-structured interviews were conducted with 15 HR professionals from the Ministry of Health and five medical universities. Participants were selected purposively based on their experience in HR management, workforce planning, and familiarity with hospital personnel processes. Interview questions focused on identifying routine duties, major work processes, task variations, and responsibilities that were not adequately reflected in existing job descriptions. Interviews continued until sufficient information was obtained to construct a preliminary list of workload components. Since job descriptions were outdated, preliminary drafts and duties were created through interviews with 15 HR professionals. Their characteristics are listed in Table 1. The duties were analyzed thematically to identify key pattern [29]. Interview transcripts and notes were analysed using thematic analysis. First, meaningful statements related to HR duties and responsibilities were identified and coded. Similar codes were then grouped into broader categories representing common work processes. Through iterative review and discussion among the research team, the codes were consolidated into major functional themes. The analysis identified four recurring functional domains across hospitals, including recruitment and staffing, personnel records and archiving, attendance and time management, and staff training and development. These themes formed the basis for developing the preliminary workload component list used in subsequent field observations and Delphi rounds.

**Table 1:** Characteristics and roles of WISN working groups.

Working Group	Participants (n)	Key roles and responsibilities
eering	University lecturers with experience in workforce planning projects and deputy directors for resources development from two medical universities (n = 4)	Methodological oversight; guidance of the technical group; monitoring adherence to WISN principles and overall study progress
Technical (Operational)	The head of the group is selected from the research team, a PhD student in Health Services Management with practical experience in WISN implementation (n = 1)	Coordination of WISN activities; Selecting, training, and guiding other operational members; preliminary data analysis.
	operational members include healthcare management students or graduates with prior internship or service experience in hospital administrative units (n = 6)	collecting data through interviews or shadow; follow-up; and collecting completed Delphi questionnaires from experts
Expert groups	First Group: Experts from the human resources and process improvement units in the Ministry of Health and five medical universities(n = 15)	Preparing job descriptions; participation in two Delphi rounds.
	Second Group: Expert working groups based on cadres, officials, and employees in human resource units from 10 public hospitals(n = 15)	Evaluation of workload components and activity standards; participation in two Delphi rounds.

### Study setting

To address challenges in documenting administrative activities, such as multitasking, irregular rotation, and gradual process completion [16,35], field study were conducted in a purposively selected public hospital. The hospital was purposively selected because it is a large public teaching hospital with an established human resources department that performs the full range of HR functions routinely found in Iranian public hospitals. In addition, the hospital maintained accessible administrative records and permitted prolonged observation of work processes. The aim was not statistical representativeness but the comprehensive identification of workload components and work processes. Because the investigated HR functions are defined according to national regulations, the resulting framework can be adapted and replicated in other public hospitals with similar organizational structures.

### WISN Working Groups

Three groups were established to support the study process, including a Steering Committee, a Technical (Operational) Group, and two Expert Groups. Their composition and responsibilities are presented in Table 1.

### Field observation and process mapping

Documenting process components and steps is recommended to ensure adherence to standard procedures and to reduce bias [21,36]. The technical (operational) group consisted of healthcare management students who had previously been introduced to the hospital environment and HR processes during their internship. They received theoretical and practical training about WISN, as well as in observation and interview methods. The training lasted with an assessment to ensure readiness. Their prior familiarity with the staff was intended to further reduce the risk of observation bias [37,38].

A baseline job description was first presented to the unit manager or designated specialist in each HR department unit to identify any missing activities. The steps in each process were documented through an interview. Approximately 200 hours of process observation were conducted, primarily through shadowing, a technique recommended for analysing office operations [39]. This technique reduces biases caused by recall during interviews and by stopwatch observations while recording work patterns in the real workplace [40–42]. Interview and shadowing reports were reviewed using three approaches:

1. Adding any missing activities to the list
2. Recording the time spent on activities
3. Review of the classification of activities of the three groups according to the information obtained from the field about the possibility of statistical reporting and the possibility of performing by most members of the job group.

### Development of workload components and activity standards

According to the WISN manual, activities that all members can perform, and their frequencies, can be extracted from systems and reports were categorized as service activities. Activities that support the execution of service tasks but lack statistics were identified as supportive activities. Other allowance activities, carried out by certain members of the cadre, were registered as additional activities [21].

Because most of HR activities are process-based and differ from routine clinical work, the observation phase aimed to establish an initial range of time estimates, rather than precise, calculated averages. If an activity was observed more than once, the average values were recorded. Initial estimates for activities not observed were obtained through structured interviews with process owners. Given that each service may include multiple components provided by employees from different departments, activity times were considered for the components provided by staff in the HR job group [43]. Time estimates were obtained using a combination of direct observation and expert judgment. For activities observed repeatedly, the duration of multiple observations was recorded and averaged. For infrequent or non-observable activities, estimates were obtained from experienced process owners and subsequently reviewed during Delphi rounds. Final activity standards were established using the median value of expert estimates to reduce the influence of extreme observations.

### Delphi consensus process

Subsequently, to finalise a valid and usable dataset, the enriched field data (including the list of activities and initial time estimates) were combined to develop a Delphi questionnaire. The observational findings, interview reports, and preliminary time estimates were systematically reviewed by the research team before Delphi administration. Activities identified through field observations were compared with interview-derived workload components, merged where appropriate, and organized according to WISN activity categories. The resulting integrated dataset served as the basis for the Delphi questionnaire, through which experts reviewed, modified, accepted, or rejected individual workload components and proposed activity standards. The instrument was validated by involving 15 WISN streering and first expert group members [44]. Closed-ended questions or practice statements were presented using a 5-point Likert Scale (strongly disagree, disagree, do not disagree or agree, agree, and strongly agree). The revised questionnaire was prepared for distribution in formal Delphi rounds and to facilitate specialist consensus. Delphi is a common structured method used to reach consensus on a workload or list of activities by ensuring fair participation and moderating dominant voices [45,46]. A concentration of 70% of the responses on points one and two of the Likert scale was considered as reaching a consensus on WC, with response stability defined as less than a 10% change between rounds [44,45]. The 70% threshold was selected based on recommendations from previous Delphi studies and WISN-related consensus research, where this level is commonly considered indicative of acceptable agreement among experts. The median was selected as the final activity standard because it is less sensitive to extreme values and provides a robust estimate when expert opinions vary.

The first round of the Delphi was conducted with 8 experts from the first group and 15 from the second group (as shown in Table 1). Key informants who ensured representation of the HR field [44,47,48]. This validation was essential for achieving consensus on adapting the standards to the local conditions of public hospitals in Iran [21]. Participants received information about the study objectives and their role in the study process and definitions of the three categories of activities along with the first round questionnaire and were asked to: 1. state their level of agreement with each WC on a Likert scale; 2. add any new activities; and 3. estimate the AS for each new or initial WC assuming based on local conditions, taking into that the employee is well trained, competent and motivated. More detailed explanations were also provided to most participants, either by telephone or in person, to help them make more informed decisions [47].

After making corrections, the questionnaire was re-examined in a second round. By providing controlled summaries and feedback on the first-round responses, participants had the opportunity to learn from others’ opinions and reconsider [47,49]. Meeting predetermined criteria for consensus and response stability marked the completion of the Delphi rounds [47,48]. The list of components and values of the time standards was finalised.

### Data analysis

Data were analysed using Excel software. Activity standards were developed using minimum, maximum, mean, and median scales to ensure consistent, agreed-upon workload components and to allow application across different scenarios [43]. To provide a more accurate estimate and reduce the impact of outliers, the median index was used as the final activity standard [50].

### Ethical considerations

Ethical approval was obtained from Tabriz University of Medical Sciences (code: IR.TBZMED.REC.1401.1023). Permission was acquired from hospital authorities before data collection. All participants were informed of the study objectives, and written informed consent was obtained before participation.

## Results

### Description of participants

Members of the WISN tripartite working groups and HR process owners from the selected hospital participated in various stages of this study. The participants’ demographic characteristics are presented in Table 2.

**Table 2:** Characteristics of participants.

	Gender		Experience		
	Female	Male	<5 years	5-15 years	>15 years
Steering Committee	1	3		1	3
Technical Group)Operational(	4	3	6	0	1
Expert Groups	14	16		3	27
Hospital cadre	4	3	0	2	5

### Phase one: Job descriptions and create baseline draft

Analysis of interviews with 15 human resources specialists from the Ministry of Health and five medical universities (Tabriz, Iran, Bushehr, Shiraz, and Maragheh) generated a list of initial workload components for consensus development. The thematic analysis resulted in the identification of four major functional domains that were consistently reported across participating organizations (recruitment and staffing processes, personnel records management and archiving, attendance and leave administration, and staff training and development activities). These domains provided the conceptual structure for organizing the initial workload components and guided subsequent field observations.

This analysis revealed the following:

1. Although hospitals affiliated with different universities had distinct organizational structures and staffing levels within the human resources department, four main functional subunits were consistently identified, including recruitment, archiving, time recording, and staff training. Personnel in these departments could be assigned to specific or shared responsibilities.
2. Job description titles were obtained as a baseline list, which served as an initial draft for the subsequent stages of the present study.

### Phase two: Field study

An analysis of reports based on shadowing observations and semi-structured interviews with the supervisor and staff of the human resources department at the selected hospital enriched and refined the initial activity draft. The process of collecting and analyzing field findings, as an example, is presented in Supplementary Table 1 in S1 File. The most important achievements of this phase are as follows:

I Completion of the activity list: Adding 13 activities to the initial list (7 service activities, 5 support activities, and 1 additional activity).
II Process segmentation and determination of implementation steps: Breaking down service activities into 246 operational steps (approximately 5.2 per activity on average) provided a more precise understanding of the workflow and enabled more accurate time estimates.
III Reallocation of activity categories: Revising the initial classification of some activities into three groups service, support, and additional by referring again to key WISN criteria, including the ability to extract statistics and perform tasks by most members of the occupational group.
IV Establishing initial time estimates: Recording the initial time range for each activity based on direct observation timing or staff estimates during interviews.

Ultimately, 47 service activities, 12 support activities, and 26 additional activities were included in the Delphi questionnaire. Pilot testing of the questionnaire resulted in minor revisions prior to formal administration. The pilot test was conducted with six HR experts who did not participate in the subsequent Delphi rounds. Based on their feedback, minor revisions were made to improve wording clarity, remove ambiguous expressions, and ensure consistency in the classification of activities before formal administration.

### Phase three: Delphi process

The panel of experts in this study included 30 health human resources specialists (Group 1 and Group 2 of the Expert, according to Table 1), of whom 24 did not participate in the pilot study and participated in both rounds of the Delphi process. Follow-up and guidance provided by the WISN Operational Team members played an effective role in preventing dropout among participants.

Forty service activity titles were agreed upon in the first round. The activities that were not agreed upon mainly involved titles that were similar or repetitive in purpose and time consumption, and these titles could be merged. Additionally, specialists suggested replacing the two similar activities with the title ‘Issuance of Corrective Decrees’.

Sample expert opinions:

*“The process of issuing internal and external organizational missions is completely similar and only differs by one or two steps; the goal is the same. If we separate such tasks, it creates multiplicity and complexity.” (E5)*

*“Issuing corrective employment decrees for war veterans is, in my opinion, a temporary activity; it may not be needed next year, as it was not required last year either.” (E2)*

*“Some activities can be described under broader, more comprehensive titles so that minor changes in expectations do not render their titles obsolete. This would help the list remain valid and sustainable across future periods.” (E30)*

The review of supportive activities in the first Delphi round resulted in the approval of 10 items (83.3%). However, items such as ‘Extraction of job file summaries’ and ‘Requesting security clearance opinions for managerial positions’ did not meet the agreement threshold because they were considered stages of other service activities. Participants also suggested adding two items: ‘Participation in monthly group meetings and follow-up of resolutions’ and ‘Daily review of the correspondence inbox’.

Of the 26 additional activities, 19 achieved agreement levels ranging from 74% to 100%**.** Two activities *‘Issuing staff identification labels’* and *‘Removing records of departing staff from the attendance system’* were identified as stages of other service activities. Consensus was not reached on ‘Updating facial recognition profiles of staff when required’; dissenting experts cited the infrequency of this activity during the year and its minimal time requirement as reasons for non-approval. For four supervisory-level duties *‘Revision and updating of the organizational structure’*, *‘Payroll system processing’*, *‘Hospital succession planning’*, and *‘Development and proposal of human resource policies and programs’* the level of agreement ranged between 56.5% and 69.6%.

Examples of comments from experts who disagreed include

*“The workload and time required for these tasks cannot be easily determined, as they are largely cognitive and dependent on thinking and judgment, without a fixed or clearly defined process. Nevertheless, the required human resources should be estimated with these considerations in mind.” (E11)*

*“Even some traditional personnel administration duties have been taken away from us, and the faculty’s human resources management office makes centralized decisions and even issues certain decrees, sending us only a copy. Under such circumstances, tasks such as succession planning and reforming the compensation system are, at least in small public hospitals like ours, not practically applicable.” (E3)*

As one human resources manager in a teaching hospital notes

*“I personally carry out these activities, but unfortunately, this is not the dominant approach in Iranian public hospitals. In recent years, this group has gradually shifted from being merely ‘personnel administration’ to ‘human resources.’ Even the lack of a comprehensive title for the group may reflect administrators’ expectations and understanding of its role.” (E1)*

After applying revisions such as removing activities that most considered part of other tasks or merging similar activities the second-round Delphi questionnaire was developed from the first-round responses. A summary of the first-round results was anonymously shared with participants.Details of the experts’ opinions during the first Delphi round are presented in three Supplementary tables 2 to Table 4 in S1 File. They were asked to express their opinions on workload components that had not reached agreement in the previous round and to propose activity standards for newly introduced workload components. Table 3 presents the consensus development process across the Delphi rounds in this study.

**Table 3:** Level of Consensus Achievement for the Three Categories of Activities Across the Delphi Rounds.

	first round	second round
service	89.4	93.5
supportive	89.9	89.9
additional	82.3	88.6
total	87.3	91.6

During the second Delphi round, among the previously non-agreed activities across the three categories, two service activities and one additional activity reached the agreement threshold. All four newly proposed activities also achieved agreement levels above 70%. Furthermore, comparison of scoring trends between the two rounds demonstrated over 90% stability in responses, indicating sufficient consensus and supporting the termination of the Delphi process.

### Phase four: Synthesis and final report of findings

During the Delphi rounds, the list of workload components and their corresponding activity time estimates was collected and analyzed in Microsoft Excel. Upon completion of the Delphi process, a final list comprising 44 service activities, 11 supportive activities, and 21 additional activities was established. Fig 2 presents the distribution of activities across the three categories.

**Fig 2:**
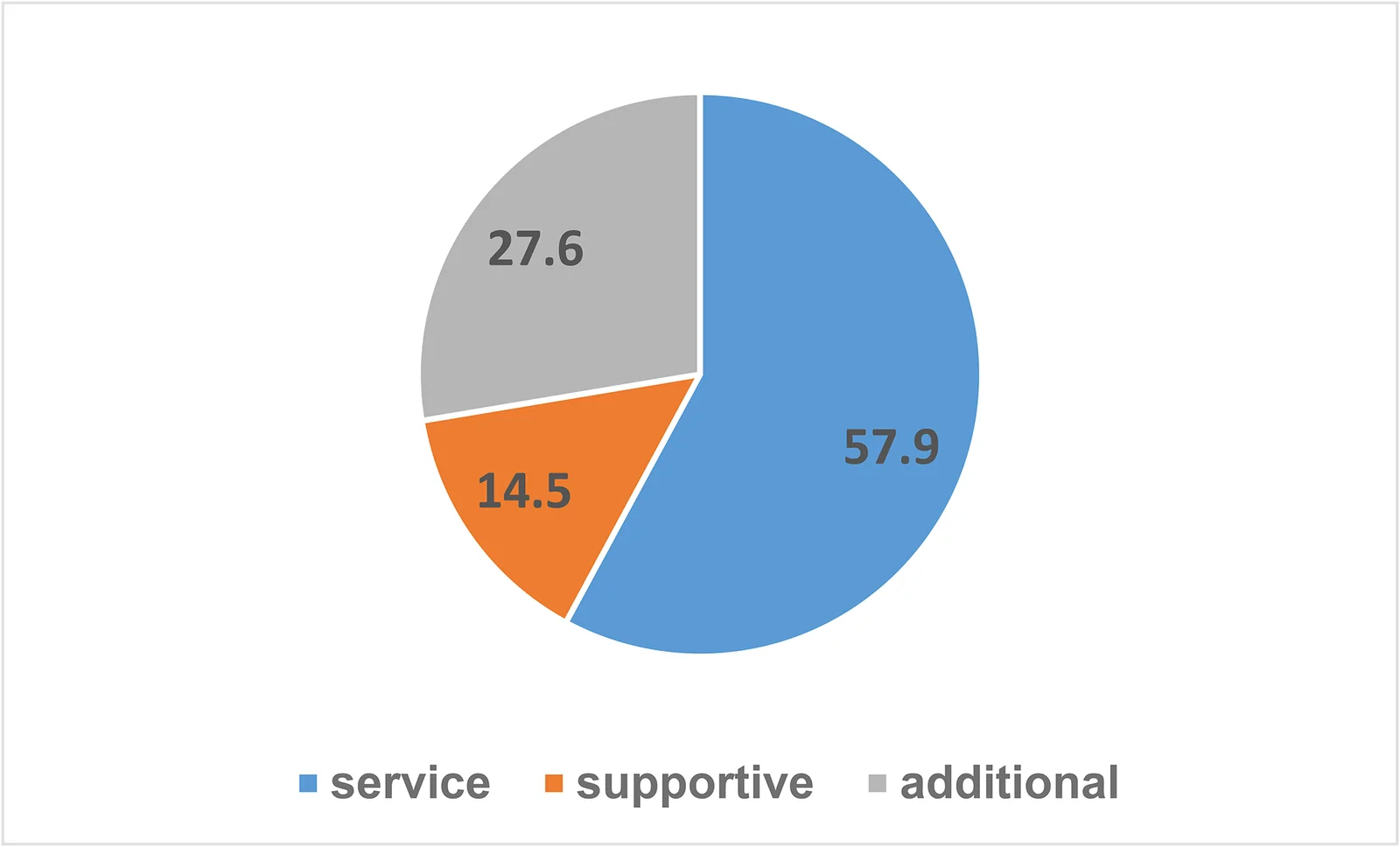
Percentage Distribution of Activities by Type.

For each activity, the level of agreement and the activity standard values, including minimum, maximum, mean, and median time, were calculated. Activities that met the ≥ 70% consensus threshold were included in the final report. A validated list of activity titles across the three categories, together with detailed time standards for each, was produced. These results provide robust empirical evidence to support subsequent staff requirement calculations based on the WISN formula (Tables 4–6).

**Table 4:** Final service activities.

Service activities		Activity standard based on the median (minutes per case)
1	Issuance of entitlement-based rank promotion decree	45
2	Issuance of entitlement-based grade promotion decree	45
3	Issuance of decree recognizing private-sector work experience	68
4	Issuance of the job title change decree	68
5	Issuance of a decree recognizing academic qualifications	60
6	Issuance of the annual salary coefficient adjustment decree	7
7	Issuance of the unpaid leave decree	40
8	Issuance of a temporary suspension decree pending disciplinary ruling	45
9	Issuance of maternity leave decree	20
10	Issuance of a part-time employment decree	20
11	Issuance of decrees based on decisions of competent legal authorities	27
12	Issuance of the hardship and shift-work allowance decree	40
13	Issuance of a decree establishing or modifying radiation allowance coefficients	16
14	Issuance of the child and family allowance decree	15
15	Issuance of special allowance decree	15
16	Issuance of administrative appointment notifications	12
17	Issuance of employment commencement decree	40
18	Issuance of decree terminating cooperation with project-based staff	27
19	Issuance of decree extending cooperation with project-based staff	30
20	Issuance of decree concluding cooperation with project-based staff	23
21	Issuance of decree authorizing continuation of education	25
22	Issuance of the inbound assignment decree	20
23	Issuance of the outbound assignment decree	30
24	Issuance of internal transfer-in decree	80
25	Issuance of internal transfer-out decree	69
26	Issuance of decree extending assignment periods	30
27	Issuance of the educational assignment decree	40
28	Issuance of the decree extending educational assignments	25
29	Issuance of the early retirement decree	64
30	Issuance of the service buyback decree	55
31	Issuance of decree establishing survivors’ benefits following employee death	70
32	Issuance of decree establishing survivors’ benefits for deceased retirees	60
33	Issuance of disability retirement decree	65
34	Issuance of retirement decree	66
35	Issuance of a contractual employment decree	30
36	Issuance of employment status conversion decree	56
37	Submission of employee misconduct reports to the Administrative Disciplinary Board	40
38	Notification of remaining leave balances to employees	5
39	Review of leave buyback requests	30
40	Review of resignation requests	60
41	Performance appraisal of hospital staff	20
42	Review of employee complaints	25
43	Issuance of corrective administrative decrees	35
44	Digitization of physical personnel files	20

**Table 5:** Final list of support activities.

Support Activities		Activity standard
1	Obtaining employee identification numbers from the “Iran Employee” system	1 h/month
2	Extracting customized performance reports based on unit requests	2h/month
3	Implementing changes to departmental shift schedules as required	1h/month
4	Proposing required training topics	1h/year
5	Providing explanations and guidance to clients/service recipients	40min/day
6	Following up on the completion of missing training hours when required	10h/year
7	Responding to telephone inquiries	30min/day
8	Communicating new work-related circulars to departments	8h/year
9	Searching for and retrieving old personnel files upon request	1h/week
10	Conducting monthly group meetings and following up on decisions	2 h/month
11	Daily review of the correspondence inbox (letter tracking system)	35 min/day

**Table 6:** Final list of additional activities.

Additional Activities		Activity standard
1	Training of student groups	14h/year
2	Review of draft letters	40min/day
3	Participation in managerial committees and meetings	2h/week
4	Participation in hospital staff–related ceremonial and special programs	3h/month
5	Preparation of performance reports	5h/month
6	Providing input on the revision of educational content and guidelines	90min/year
7	Collaboration in research activities	1 h/month
8	Responding to administrative staff inquiries	1 h/month
9	Collaboration with accreditation and audit teams	5h/year
10	Approval of off-site work missions for staff	1 h/month
11	Orientation of newly recruited hospital staff to regulations and the work environment	6 h/month
12	Preparation of guidelines and procedures for improving hospital staff performance	5 h/year
13	Obtaining authorization for the employment of project-based staff	1 h/month
14	Monitoring indicators related to staff health	6 h/year
15	Adjusting work schedules based on officially communicated changes	4 h/year
16	Destruction of expired documents	4 h/year
17	Development and proposal of human resources programs and policies	10 h/year
18	Scheduling and coordination of the human resources group meetings	30 min/month
19	Handling hospital staff disciplinary issues	8 h/year
20	Physical cleaning and organization of personnel files	4 h/month
21	Compilation of documentation and reports related to training courses	10 h/ year

## Discussion

This study provides one of the first validated, evidence-based frameworks for determining workload components and activity standards for human resource staff in public hospitals. It includes experience adapting the key steps of WISN in a non-clinical setting, addresses the unique challenges of determining work components and activity standards for the sector, and highlights strategic considerations, proposing a structured approach to achieve consensus on the transparency and validity of WISN’s application in non-clinical positions.

A combination of methods was used to address potential biases and increase the accuracy and reliability of the data [21]. Primary data were obtained through interviews with national and university experts and were reinforced and supplemented with field information. The baseline list was revised and finalized through two rounds of the Delphi method. This integrated approach aligns with efforts to address limitations in estimating workforce needs, including those for doctors and nurses [29,51]. The agreement achieved in the second round of Delphi was 91.6. Comparable levels of agreement have been reported in previous Delphi‑based workforce studies [44]. This result can be attributed to the enriched questionnaire input obtained during face-to-face meetings with experts and field validation. This approach is consistent with key recommendations in the Delphi literature, which emphasize providing participants with rich, multidimensional background information and combining the Delphi technique with other qualitative methods to yield stronger results [47,52].

This study shows a distinct distribution of service, support, and additional activities compared with clinical areas, which report a narrower range of activity types and a larger share of service activities [14,53,54]. This difference may reflect the nature of work in nonclinical support areas, such as human resources, which are characterized by tasks that are intertwined, multi-layered, indirect, and process-oriented. In some cases, the routine activities of one subgroup, such as timekeeping, may simultaneously support the primary mission of another subgroup (such as recruitment). Also, some of the archival subgroup’s tasks, which are not yet easily measurable within traditional frameworks, are classified as additional activities. The comprehensive approach adopted in this study, which considers the interrelationships among subgroups to map activities at the departmental level, can help clarify the actual workload and yield different outcomes than approaches that examine each administrative subgroup separately [55]. The Iterative process of defining workload components revealed that, at times, demarcating the three activities according to the WISN definitions can be challenging or ambiguous [56]. In this study, this challenge was addressed through accurate reallocation during field studies and data enrichment. This challenge in classifying activities is also observed in other studies. For example, the activity of “patient reporting” has been placed in different categories in studies depending on the researcher’s opinion [21,43,44]. Similar ambiguity has been observed among clinical personnel, including nurses [31], ndicating that this challenge is not unique to non-clinical positions. Identifying and addressing this ambiguity in this study could help improve the accuracy of the WISN application. It also highlights the need for a clear, operational redefinition of activities, especially in non-clinical positions.

Similar efforts to define workload components and activity standards have been reported in South Africa, Ghana, Oman, and several WHO African Region countries using the WISN methodology [20,30,31,42]. Consistent with these studies, our findings highlight the importance of establishing locally validated workload components before workforce requirements are calculated. However, unlike most previous studies that focused on clinical occupations, the present study addresses a non-clinical professional group and demonstrates the additional challenges associated with administrative, cognitive, and process-oriented activities.

The key findings of this study indicate a gap between the expected and perceived roles of HR departments. While national and academic experts expect HR heads to play an active role in strategic activities such as designing incentive programs or succession planning, HR professionals generally do not see themselves in these roles and often remain in traditional administrative functions. This gap could reflect an incomplete and evolving transition from the conventional concept of “recruitment” to the more comprehensive concept of “human resources.”Another factor contributing to the lack of agreement on strategic hospital HRM activities was the nature of “invisible work” and the challenge of measuring its volume. Similar patterns have been reported in previous studies [54], which found that a weak, traditional understanding of HR professionals leads to a disregard for their capacity to address strategic challenges. Similarly, Mamata et al. [19] have highlighted that this occupational group remains deprived of effective role development despite numerous reforms over recent decades. A clear definition of the required competencies, as provided by Zhang and Chen for the roles of strategist, change agent, administrative specialist, and employee champion, can be practical for navigating this transition [17]. In fact, this gap is not simply a management issue. Instead, it is a structural challenge that requires coordinated action at the micro, meso, and macro levels to resolve [9].

Considerable variation was identified in the duration of additional activities and some service activities. This indicates that administrative processes need to be improved and standardized. The origin of this variation can be traced to the evolving and less mechanized nature of some activities. For example, the activity of “issuing employee pension orders” typically takes longer to verify insurance records for employees covered by traditional insurance systems. Similar findings have been reported in clinical settings, where administrative efficiency has been improved by enabling electronic communication and networking between organizations [57]. However, as Poisson et al. caution, digitalization does not necessarily guarantee a reduction in process time. Without strategic implementation, electronic workflows can unintentionally lengthen activity durations [58]. From this perspective, the framework involves reporting minimum, maximum, median, and average values for each activity, an approach successfully used in previous studies [22,43] that allows for more realistic and consistent estimates of human resources across different scenarios.

### Implications for policy and practice

The findings of this study have important practical and policy implications. The framework developed in the study strengthens evidence-based workforce planning by providing a reliable list of workload components and performance standards for HR functions, moving beyond anecdotal opinions and traditional approaches. It also provides a valid framework for obtaining more accurate data from steps three and four of the WISN method, especially in areas where the initial list is neither valid nor agreed upon. Policy changes at the macro to operational levels are necessary to strengthen the HR department’s position and enable employees to play the expected and specialized roles in this complex.

## Conclusion

This study is the first to address the research gap in the application of WISN in the non-clinical positions of Iranian hospitals. The use of a structured framework and a triangular approach enabled a high level of consensus in defining workload components and activity standards for human resource staff. This study provided a picture of the job descriptions and activity distribution patterns, as well as the scope of support and additional activities. The resulting standards, rather than being purely theoretical or limited to specific contexts, are determined by a careful integration of empirical measurement and professional judgment. This study, while identifying measurable administrative tasks, emphasizes the existence of strategic tasks in human resource departments that are inadequately addressed and invisible.

### Limitation

Several limitations should be kept in mind in regard to the results. First, the field observations were in one public hospital, even though multiple hospitals and university experts were in the consensus step. Second, the existing digitalization, organizational and administrative frameworks of the Iranian public hospitals influenced the activity standards. Third, some of the strategic and cognitive HR activities were hard to observe and were evaluated indirectly based mainly on the judgment of the experts. Finally, changes in administrative technologies or national policies may require future updates to the proposed activity standards.

## Supporting information

S1 File Supporting tables for the study. (DOCX)

## Abbreviation:

HR: human resources
WISN: Workload Indicators of Staff Need
WHO: World Health Organization
WC: workload components
AS: activity standards
