# Supplementary material for: Defining workload components and activity standards for human resources professionals in Iranian public hospitals: A WISN-based framework for strengthening non-clinical workforce planning
Source: PLoS One. 2026 Oct 8;21(10):e0356051. doi: 10.1371/journal.pone.0356051 (PMC13648983; doi:10.1371/journal.pone.0356051)
Supplement: Supplementary file 1 [file pone.0356051.s001.docx]

**S Table1.** Sample of data collection and analysis of shadowing observations and interviews with process owners

| **Data collection method** | **Participant Profile** | **Initial reporting and analysis** | **Proposed results for WC/AS** |
| --- | --- | --- | --- |
| Shadowing | Ms. R. R.: Personnel affairs officer,  26 years of total work experience,  15 years of experience in personnel affairs. | At 8:00 a.m., we arrived at the workplace of Ms. R.R., a personnel affairs officer at the hospital. She turned on her computer system. First, she reviewed the cases of clients remaining from the previous day and addressed the deficiencies in three files. She asked her colleague, Mr. M.T., to obtain family allowance information for a ward nurse, Ms. S.M., by phone so that the personnel record could be updated. She then noted the required follow-up actions for the remaining files.  At 8:30 a.m., three clients (hospital staff from different departments) arrived simultaneously—two nurses and one staff member from the emergency department. She referred one of them to Mr. S.R. (staff training officer). Regarding one of the nurses, based on the personnel file number, she explained that the responsible officer was Mr. F.A., another personnel affairs expert in Room 2, and provided guidance accordingly. For the third client, a nurse nearing retirement, she asked several questions and requested that the individual retrieve their paper file from the archive and submit it for review. She reassured the client that they would be informed of the progress by phone. At 9:00 a.m., as they were about to have breakfast, I left the room.  Around 9:30 a.m., when I returned, Ms. R.R. was speaking on the office telephone, during which her personal mobile phone also rang. She left the room and spoke for about ten minutes. After returning, her colleagues asked questions about how to apply the coefficient for physicians’ work experience, and she provided explanations.  At 10:10 a.m., she began reviewing physical files whose information had not yet been updated in the electronic system and entered the required data into the system. She continued this task at a good pace, except in cases where she needed to ask her unit supervisor for additional information. Approximately six files were completed within about one hour.  At 11:20 a.m., health management internship students entered the office (it was their third day in this unit). After reviewing the topics from previous days, they opened page 9 of the workbook and asked questions about different types of organizational correspondence. Ms. R.R., together with the unit supervisor who was present in the office, provided thorough explanations (for 60 minutes). During this time, three clients also visited, and … | Updating file information took approximately 10 minutes.  During the day, there were 6 in-person visits, and responding to each visitor took an average of 10 minutes.  For training internship students, training is conducted twice a year, each time for 6 days, with approximately 60 to 90 minutes spent per day. |
| Interview | Mr. N.A.  Head of the Hospital Human Resources Department, with 25 years of total work experience and 16 years of experience in various HR units. | Question: *Is there any activity that has not been included in this list?*  Answer:  *“Sometimes a large portion of our time in the personnel (HR) office is spent on informing employees about laws and regulations. Someone working in a department may not be familiar with the relevant rules and regulations, but the human resources department must support them so they are not harmed or disadvantaged. We have a duty to protect our colleagues. For example, an employee who does not know that holding two jobs is an offense may work in two places and even face dismissal. This is one of the key responsibilities of this professional field. There is no place where statistics for this are recorded, but almost all of us are involved in this important duty. Or consider someone working in a clinical unit who does not realize that their work involves certain risks; the human resources department must take these issues into account and support employees against such risks. In short, providing employees with informational support about protective laws and regulations is a very important role that is often overlooked.”* | Adding the supportive activity “Informing employees about laws and circulars” to the initial list. |

**S Table 2. Detailed results of the first Delphi round: service activities**

|  | | **WC Results of Review and Aggregation** | | | | **AS Results of Review and Aggregation** | | | | | |
| --- | --- | --- | --- | --- | --- | --- | --- | --- | --- | --- | --- |
| **Service Activities** | | **Agree & Strongly Agree** | **Neutral** | **Disagree & Strongly Disagree** | **Overall Agreement (%)** | **Mean** | **Mode** | **Minimum** | **Maximum** | **Median** | **Field study** |
| 1 | Issuance of entitlement-based rank promotion decree | 23 |  |  | 100.0 | 44.0 | 40 | 25 | 65 | 42 | 47 |
| 2 | Issuance of entitlement-based grade promotion decree | 23 |  |  | 100.0 | 44.0 | 30 | 25 | 65 | 35 | 37 |
| 3 | Issuance of decree recognizing private-sector work experience | 23 |  |  | 100.0 | 66.8 | 65 | 25 | 75 | 60 | 68 |
| 4 | Issuance of the job title change decree | 15 | 4 | 4 | 65.2 | 66.8 | 60 | 40 | 65 | 58 | 68 |
| 5 | Issuance of a decree recognizing academic qualifications | 23 |  |  | 100.0 | 58.3 | 50 | 35 | 100 | 56 | 56 |
| 6 | Issuance of the annual salary coefficient adjustment decree | 20 | 3 |  | 87.0 | 8.4 | 7 | 5 | 10 | 7 | 7 |
| 7 | Issuance of the unpaid leave decree | 23 |  |  | 100.0 | 42.2 | 35 | 15 | 76 | 39 | 39 |
| 8 | Issuance of a temporary suspension decree pending disciplinary ruling | 18 | 3 | 2 | 78.3 | 42.6 | 40 | 40 | 80 | 47 | 47 |
| 9 | Issuance of maternity leave decree | 23 |  |  | 100.0 | 19.1 | 15 | 10 | 25 | 16 | 16 |
| 10 | Issuance of a part-time employment decree | 23 |  |  | 100.0 | 25.0 | 18 | 15 | 30 | 20 | 20 |
| 11 | Issuance of decrees based on decisions of competent legal authorities | 19 | 2 | 2 | 82.6 | 31.0 | 25 | 25 | 36 | 27 | 27 |
| 12 | Issuance of the hardship and shift-work allowance decree | 23 |  |  | 100.0 | 41.1 | 40 | 40 | 52 | 42 | 42 |
| 13 | Issuance of a decree establishing or modifying radiation allowance coefficients | 18 | 5 |  | 78.3 | 16.7 | 13 | 13 | 31 | 16 | 16 |
| 14 | Issuance of the child and family allowance decree | 23 |  |  | 100.0 | 15.4 | 15 | 10 | 21 | 15 | 15 |
| 15 | Issuance of special allowance decree | 23 |  |  | 100.0 | 17.2 | 12 | 8 | 15 | 12 | 12 |
| 16 | Issuance of administrative appointment notifications | 23 |  |  | 100.0 | 13.1 | 7 | 5 | 10 | 7 | 7 |
| 17 | Issuance of employment commencement decree | 23 |  |  | 100.0 | 45.7 | 45 | 30 | 117 | 55 | 55 |
| 18 | Issuance of decree terminating cooperation with project-based staff | 23 |  |  | 100.0 | 25.3 | 25 | 23 | 37 | 27 | 27 |
| 19 | Issuance of decree extending cooperation with project-based staff | 23 |  |  | 100.0 | 29.8 | 25 | 25 | 45 | 28 | 28 |
| 20 | Issuance of decree concluding cooperation with project-based staff | 23 |  |  | 100.0 | 22.6 | 22 | 20 | 32 | 23 | 23 |
| 21 | Issuance of decree authorizing continuation of education | 23 |  |  | 100.0 | 25.8 | 24 | 18 | 36 | 25 | 25 |
| 22 | Issuance of the inbound assignment decree | 20 | 3 |  | 87.0 | 20.9 | 15 | 10 | 27 | 16 | 16 |
| 23 | Issuance of the outbound assignment decree | 20 | 3 |  | 87.0 | 32.0 | 22 | 10 | 70 | 28 | 28 |
| 24 | Issuance of internal transfer-in decree | 20 | 3 |  | 87.0 | 77.4 | 75 | 40 | 220 | 93 | 93 |
| 25 | Issuance of internal transfer-out decree | 20 | 3 |  | 87.0 | 63.9 | 60 | 55 | 120 | 69 | 69 |
| 26 | Issuance of external transfer-in decree | 15 | 6 | 2 | 65.2 | 87.4 | 110 | 75 | 218 | 122 | 122 |
| 27 | Issuance of external transfer-out decree | 15 | 6 | 2 | 65.2 | 72.0 | 75 | 75 | 120 | 83 | 83 |
| 28 | Issuance of decree extending internal assignment periods | 20 | 3 |  | 87.0 | 30.0 | 30 | 20 | 40 | 30 | 30 |
| 29 | Issuance of decree extending external assignment periods | 15 | 6 | 2 | 65.2 | 30.2 | 30 | 20 | 40 | 30 | 30 |
| 30 | Issuance of the educational assignment decree | 20 | 3 |  | 87.0 | 37.9 | 40 | 40 | 60 | 43 | 43 |
| 31 | Issuance of the decree extending educational assignments | 19 | 4 |  | 82.6 | 26.8 | 25 | 25 | 30 | 26 | 26 |
| 32 | Issuance of the early retirement decree | 20 | 3 |  | 87.0 | 62.0 | 55 | 50 | 115 | 64 | 64 |
| 33 | Issuance of the service buyback decree | 20 | 3 |  | 87.0 | 51.3 | 35 | 35 | 65 | 40 | 40 |
| 34 | Issuance of decree establishing survivors’ benefits following employee death | 20 | 3 |  | 87.0 | 70.8 | 60 | 60 | 110 | 68 | 68 |
| 35 | Issuance of decree establishing survivors’ benefits for deceased retirees | 20 | 3 |  | 87.0 | 56.7 | 50 | 35 | 65 | 50 | 50 |
| 36 | Issuance of disability retirement decree | 21 | 2 |  | 91.3 | 66.6 | 65 | 55 | 110 | 71 | 71 |
| 37 | Issuance of retirement decree | 20 | 3 |  | 87.0 | 62.8 | 45 | 15 | 200 | 66 | 66 |
| 38 | Issuance of the contractual employment decree | 23 |  |  | 100.0 | 30.3 | 30 | 20 | 45 | 31 | 31 |
| 39 | Issuance of employment status conversion decree | 23 |  |  | 100.0 | 53.9 | 50 | 35 | 100 | 56 | 56 |
| 40 | Submission of employee misconduct reports to disciplinary authorities | 20 | 3 |  | 87.0 | 40.9 | 20 | 15 | 50 | 24 | 24 |
| 41 | Notification of employee leave balance status | 23 |  |  | 100.0 | 5.5 | 3 | 2 | 5 | 3 | 3 |
| 42 | Review and processing of leave buyback requests | 23 |  |  | 100.0 | 33.9 | 28 | 18 | 50 | 30 | 30 |
| 43 | Review and processing of resignation requests | 23 |  |  | 100.0 | 59.4 | 50 | 37 | 100 | 56 | 56 |
| 44 | Issuance of a corrective retirement decree | 14 | 3 | 6 | 60.9 | 26.7 | 25 | 16 | 40 | 26 | 26 |
| 45 | Issuance of the decree changing the employment status of veterans | 14 | 4 | 5 | 60.9 | 39.2 | 30 | 25 | 75 | 37 | 37 |
| 46 | Conduct of hospital staff performance appraisals | 23 |  |  | 100.0 | 21.6 | 18 | 12 | 25 | 18 | 18 |
| 47 | Review and handling of employee complaints | 17 | 3 | 3 | 73.9 | 25.4 | 35 | 20 | 20 | 30 | 46 |
| 48 | Issuance of corrective administrative decrees | Proposal to merge items 44 and 45. | | | |  |  |  |  |  |  |

**S Table 3. Detailed results of the first Delphi round: support activities**

| **Support activities** | | **Strongly agree/agree** | **Do not agree/ disagree** | **Disagree/ strongly disagree** | **Agreement percentage** | **AS (Mean)** |
| --- | --- | --- | --- | --- | --- | --- |
| 1 | Obtaining employee identification numbers from the “Iran Employee” system | 23 |  |  | 100.0 | 1h/month |
| 2 | Extracting customized performance reports based on unit requests | 18 | 5 |  | 78.3 | 2h/month |
| 3 | Coordinating with department supervisors to approve shift schedules and leaves | 23 | 0 |  | 100.0 | 1h/month |
| 4 | Proposing required training topics | 19 | 4 |  | 82.6 | 1h/year |
| 5 | Providing explanations and guidance to clients/service recipients | 23 | 0 |  | 100.0 | 40min/day |
| 6 | Following up on the completion of missing training hours when required | 17 | 6 |  | 73.9 | 10h/year |
| 7 | Responding to telephone inquiries | 23 | 0 |  | 100.0 | 25 min/day |
| 8 | Communicating new work-related circulars to departments | 22 | 1 |  | 95.7 | 8h/year |
| 9 | Extracting summaries of personnel employment records | 15 | 4 | 3 | 65.2 | 30 min/day |
| 10 | Requesting security clearance opinions for managerial positions | 15 | 4 | 3 | 65.2 | 1 h/month |
| 11 | Digitization of physical personnel files | 15 | 4 | 3 | 65.2 | 6 h/month |
| 12 | Searching for and retrieving old personnel files upon request | 18 | 2 | 3 | 78.3 | 1h/week |
| 13 | Holding weekly group meetings and following up on decisions | Proposed | | | | |
| 14 | Daily review of the correspondence inbox (letter tracking system) | Proposed | | | | |

**S Table 4. Detailed results of the first Delphi round: additional activities**

|  | | **Number of strongly agree/agree responses** | **Neutral** | **Disagree/**  **strongly disagree** | **Agreement percentage** | **Median** |
| --- | --- | --- | --- | --- | --- | --- |
| 1 | Student group training | 17 | 6 |  | 73.9 | 420 |
| 2 | Review of draft letters | 22 |  |  | 95.7 | 40 |
| 3 | Participation in committees and coordination meetings | 22 |  |  | 95.7 | 120 |
| 4 | Participation in staff-related occasion programs | 22 |  |  | 95.7 | 180 |
| 5 | Preparation of performance reports | 20 | 3 |  | 87.0 | 300 |
| 6 | Providing feedback on the preparation and revision of educational content and SOPs | 18 | 5 |  | 78.3 | 90 |
| 7 | Collaboration in research activities | 19 | 4 |  | 82.6 | 60 |
| 8 | Responding to administrative inquiries | 22 |  |  | 95.7 | 60 |
| 9 | Collaboration with accreditation and audit teams | 23 |  |  | 100.0 | 300 |
| 10 | Approval of off-site staff assignments | 23 |  |  | 100.0 | 60 |
| 11 | Orientation of new hospital staff to regulations and work environment | 23 |  |  | 100.0 | 90 |
| 12 | Preparation of guides and SOPs for staff performance improvement | 22 |  |  | 95.7 | 150 |
| 13 | Obtaining authorization for project-based staff employment | 18 | 4 | 1 | 78.3 | 60 |
| 14 | Monitoring staff health-related indicators | 19 | 4 |  | 82.6 | 180 |
| 15 | Issuing ID badges for staff | 15 | 5 | 3 | 65.2 | 10 |
| 16 | Removing outgoing staff records from the attendance system | 15 | 5 | 3 | 65.2 | 10 |
| 17 | Updating staff facial records as needed | 15 | 5 | 3 | 65.2 | 12 |
| 18 | Adjusting work hours for women under the reduced-hour law | 18 | 2 | 3 | 78.3 | 12 |
| 19 | Destruction of expired documents | 17 | 4 | 2 | 73.9 | 240 |
| 20 | Drafting and proposing HR programs and policies | 16 | 5 | 2 | 69.6 | 600 |
| 21 | Scheduling and coordinating HR group meetings | 17 | 6 | 0 | 73.9 | 30 |
| 22 | Handling staff disciplinary matters | 23 |  |  | 100.0 | 35 |
| 23 | Physical cleaning and organization of files | 23 |  |  | 100.0 | 45 |
| 24 | Revising and updating organizational structure | 13 | 6 | 4 | 56.5 | 240 |
| 25 | Payroll system processing | 15 | 4 | 4 | 65.2 | 240 |
| 26 | Hospital succession planning | 15 | 4 | 4 | 65.2 | 240 |
| 27 | Summarizing documentation and training course reports | Proposed | | | |  |
